# Must a Knee with Anterior Cruciate Ligament Deficiency and High-grade Pivot Shift Test Present an Increase in Internal Rotation? *

**DOI:** 10.1055/s-0044-1779316

**Published:** 2024-03-21

**Authors:** Luiz Henrique Pires de Lima, João Luiz Ellera Gomes

**Affiliations:** 1 Universidade Federal do Rio Grande do Sul, Porto Alegre, RS, Brasil

**Keywords:** anterior cruciate ligament, joint instability, biomechanical phenomena

## Abstract

**Objective:** Rupture of the anterior cruciate ligament (ACL) is one of the most common injuries in athletes and is often associated with damage to anterolateral structures. This combination of injuries presents itself clinically as a high-grade pivot shift test. The hypothesis of this study is that patients with ACL deficiency and high-grade pivot shift test should have an increased internal knee rotation.

**Methods:** Twenty-two patients were tested. After effective spinal anesthesia, two tests were performed with the patient in supine position. First, the bilateral pivot shift test was performed manually, and its grade was recorded. Then, with the knee flexed to 90 degrees, the examiner drew the projection of the foot in a neutral position and in maximum internal rotation, and the angle of internal rotation was measured from the axes built between the central point of the heel and the hallux.

**Results:** In the ACL-deficient knee, it was observed that there is a statistically significant average internal rotation (IR) delta of 10.5 degrees between the groups when not adjusted for age, and 10.6 degrees when adjusted for age.

**Conclusions:** Knees with ACL deficiency and with pivot shift test grade I do not show increased internal rotation in relation to knees with intact ACL. Knees with ACL deficiency and with pivot shift test grades II and III show increased internal rotation in comparison to healthy knees.

## Introduction

Rupture of the anterior cruciate ligament (ACL) is one of the most common injuries in athletes. 1 Its reconstruction has been one of the most performed orthopedic procedures and it has been showing some postoperative complications, such as unsatisfactory rotational control, with a success rate ranging from 69 to 95%. 2 3 This inadequate restoration of biomechanics results in a significant number of patients who do not return to their previous level and/or type of physical activity 4 and have an increased risk of a new ligament failure. 5

As demonstrated by Terry et al., 6 in the vast majority of knees with an ACL rupture, there is also injury to lateral structures associated with the iliotibial tract (ITT). The anterolateral ligament (ALL) acts on the rotational stability of the knee with an ACL injury 7 ; therefore, the associated injury of these ligaments leads to an increase in the internal rotation (IR) of the knee and the degree of the pivot shift (PS), 8 and it is also pointed out as a cause of ACL reconstruction failures. 8

The positive PS test is an important indicator of rotational instability in the knee. 9 Some meta-analyses have shown a high rate of patients with a persistently positive result in the PS test in the postoperative period. 10 11 12 In a systematic review, Mohtadi suggests a 19% prevalence of grade-II or higher PS after an ACL reconstruction 11 ; other studies show that this test remains positive in more than 30% of the cases and that this instability leads to secondary lesions of the meniscus and cartilage. 13 14

Several cadaver studies have shown that, if there is a concomitant ACL injury and anterolateral structures, there will be an increase in the PS degree and passive IR of the knee at flexion angles greater than 30°; 8 however, there is a lack of clinical studies that explore these two tests together. The present study aims at correlating the degree of PS and knee IR in patients with an ACL injury. The PS test is subjective and does not have good accuracy or reproducibility. 15 The measurement of knee IR at 90° of flexion is an objective test of the physical examination and, therefore, has a probable low intra and interobserver variability. Thus, we intended to establish a more reliable clinical test, with easier reproducibility and standardization for the diagnosis of anterolateral rotational instability of the knee.

The hypothesis of this study is that patients with an ACL deficiency and a high grade in the PS test should have an increased IR of the knee.

## Methods

### Cross-sectional Study

All procedures performed in studies involving human participants were in accordance with the ethical standards and was approved by the research ethics committee of Hospital de Clínicas de Porto Alegre (CAAE number 09548118.9.0000.5327). For the sample calculation, we conducted a pilot study with a significance level of 5%, power of 90%, and an effect size of 1.5 standard deviations (SDs) between groups, obtaining a minimum of 10 patients per group. There was a mean difference in IR of 8.5 between the injured knee and the healthy knee between groups I (PS 1; IR mean = 9.83, SD = 3.25, n = 6) and groups II (PS 2 and 3; mean = 1.33, SD = 2.06, n = 6).

Among patients with complete bone maturity and with a chronic ACL deficiency who would undergo ligament reconstruction in Porto Alegre, RS, Brazil, between August 2019 and September 2020, 22 were tested. No knee had posterior meniscal root injuries or meniscal ramp lesion. Of the 22 patients included in the study, 15 had meniscal injuries (68.18%). Of these 15 patients, 6 were assigned to group II: 3 with injury to the lateral meniscus, 1 with injury to the medial meniscus, 1 with injury to both menisci, and 1 with injury to the lateral meniscus + grade-IV chondral injury smaller than 1 cm ^2^ . In group I, 9 patients had meniscal injuries: 4 with injuries to the lateral meniscus, 4 with injuries to the medial meniscus, and 1 with injuries to both menisci.

**Fig. 1:**
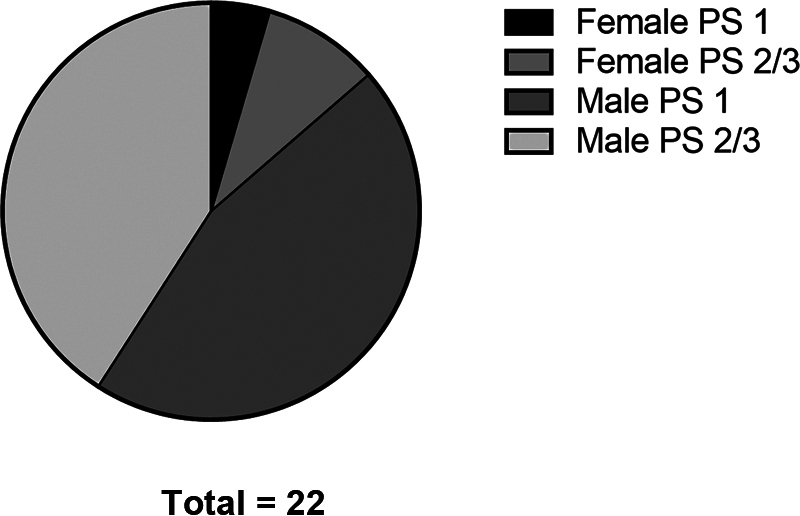
Proportion of females and males in groups I and II.

Eleven patients (1 female and 10 males) with grade-I pivot shift were allocated to group 1, whereas 6 patients with grade-II pivot shift and 5 patients with grade-III pivot shift were allocated to group 2 (2 females and 9 males) ( Fig. 1 ). The mean age of the patients was 22 years. The minimum range of motion expected for all knees was between 0° and 130°. A questionnaire about age, sex, time of injury, presence of any systemic disease, or injury to the lower limbs was answered. Patients with a history of previous injury, surgery or neurological pathologies of the knee or lower limb, locked knee, rheumatoid disease or other inflammatory disease of the joints, congenital lower limb malformation that could influence the rotation of the leg or foot, or significant arthrosis, were excluded.

**Fig. 2:**
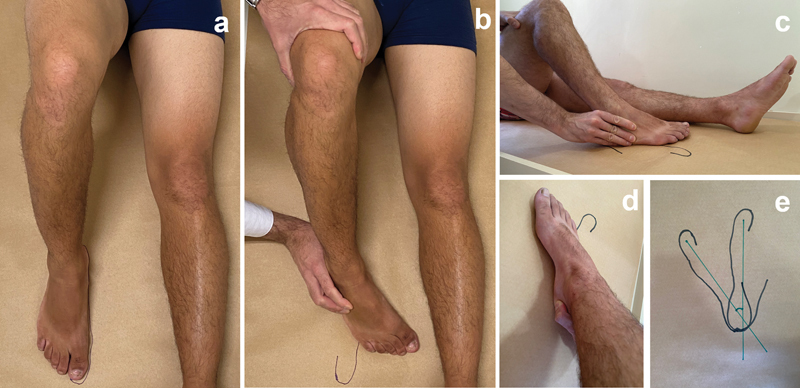
Internal rotation protocol: a: foot in a neutral position; b: foot in maximum internal rotation; c: fingers pressing the lateral face of the base of the 5th metatarsal; d: thumb making a fulcrum on the medial face of the calcaneus; e: axes built between the central point of the heel and the hallux (foot in a neutral position and in maximum internal rotation).

After spinal anesthesia, two tests were performed with the patients in the supine position. First, the bilateral pivot shift test was performed manually, and its grade was recorded according to the classification determined by the International Knee Documentation Committee (IKDC) 2000. 16 Then, while an assistant kept the patient's thigh immobilized while the knee was flexed at 90° and the foot was supported on a rigid table, the examiner drew the projection of the foot in a neutral position and in maximum internal rotation, turning the heel over its own axis, using the thumb to make a fulcrum on the medial face of the calcaneus, and using the other fingers to press the lateral face of the base of the 5th metatarsal ( Fig. 2 ). The angle of internal rotation was measured from the axes built between the central point of the heel and the hallux. The maximum internal rotation was obtained when the increased load did not generate more movement.

All tests were performed bilaterally by the same examiner while the patient was anesthetized, following the same protocol for all the patients and before any surgical incision. In each patient, the internal rotation measured on their knee without injury was considered normal.

### Statistical Analyses

The variables were described by means and standard deviations. To compare means, the Student t-test was applied. To control the effect of age, the analysis of covariance (ANCOVA) was used. The level of significance adopted was 5% ( *p* < 0.05), and the analyses were performed using the IBM SPSS Statistics for Windows, version 21.0 software (IBM Corp., Armonk, NY, USA).

## Results

**Fig. 3:**
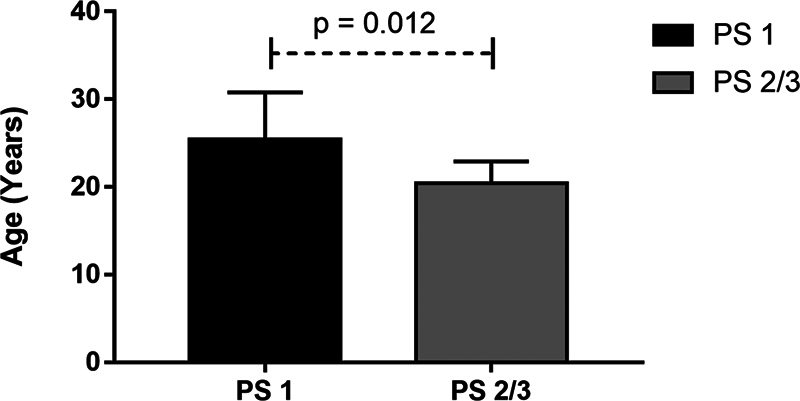
Average age of participants in groups I (PS 1) and II (PS 2 and 3).

The average age among participants was 22, with 25.4 in group 1 and 20.4 in group 2. The youngest participant was 16 years old and the oldest was 36 years old. Group 1 had a significantly higher mean age than group 2 (25.4 ± 5.4 vs 2.4 ± 2.5; *p* = 0.012) ( Fig. 3 ). Among the 22 participants, 3 were female and 19 were male ( Fig. 1 ). The mean IR of the knees with preserved ACL was 30.72° (18–48°; n = 22). The mean IR of the knees with ACL injuries in group 1 was 30° (21–47°; n = 11). The mean IR of the knees with ACL injuries in group 2 was 43.36° (36–54°; n = 11).

**Fig. 4:**
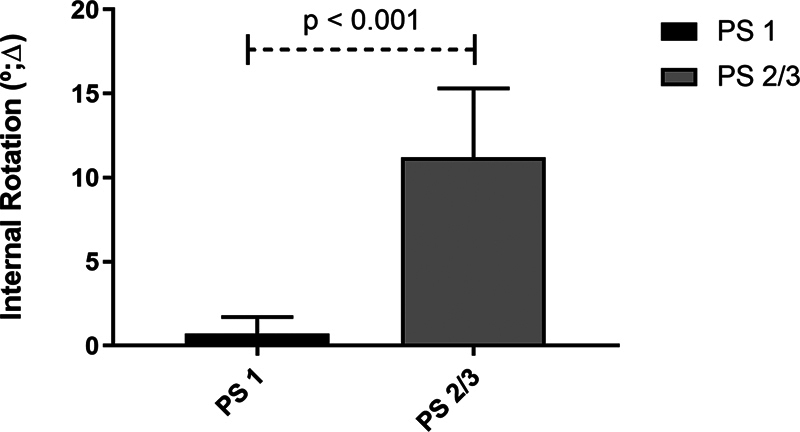
Average of internal rotation of participants in groups I (PS 1) and II (PS 2 and 3).

Table 1 and Fig. 4 show the comparison between the groups regarding the IR delta. We observed an average difference of 10.5° between the groups when not adjusted for age, and 10.6 ° when adjusted for age. In both conditions, the differences were statistically significant ( *p* < 0.001).

**Table 1:** Comparison between the two groups regarding the knee internal rotation delta

	Average ± SD		Difference (CI 95%)	*p* -value	Adjusted difference * (CI 95%)	*p* -value
	PS 1	PS 2/3				
**Delta IR (°)**	0.7 ± 1.0	11.2 ± 4.1	10.5 (7.8–13.1)	< 0.001	10.6 (7.3–13.8)	< 0.001

Abbreviations: CI, confidence interval; IR, internal rotation; SD, standard deviation.

* Adjusted for age by the analysis of covariance (ANCOVA).

## Discussion

Our results confirm the primary hypothesis: in a knee with ACL deficiency and greater anterolateral instability (with PS grade II or III), there was an increase in IR in relation to the contralateral knee with an intact ACL. On the other hand, in a knee with ACL deficiency and lesser anterolateral instability (with PS grade I), there was no increase in IR in relation to the contralateral knee with an intact ACL.

The purpose of the study was to compare the increase in IR between the knees of the PS 1 group and the PS 2 or 3 group. It was found that patients with PS 2 or 3 have a greater increase in IR than patients with PS 1. Kinematics presented different changes in rotational stability between the groups studied, corroborating our hypothesis. Therefore, the increase in internal knee rotation is of significant clinical importance.

The first researchers to point out the importance of the anterolateral ligament (ALL) were Claes et al., 17 who came to the conclusion that when the knee has a flexion angle greater than 35°, the ALL has great importance as a stabilizer of the IR. They also observed that with increasing flexion the ACL has less involvement in this function. Rasmussen et al. demonstrated that the ACL/ALL-deficient state resulted in significant increases in the static IR as well as in the axial plane translation and the IR during simulated PS when compared with the intact and ACL-deficient states at all flexion angles. The ACL-deficient state resulted in significant increases in the IR from 0 to 45° of knee flexion versus the intact state. 18 Bonanzinga et al. 5 showed that the ALL plays a significant role in controlling the static IR and PS in the setting of an ACL-deficient knee. Monaco et al. 14 demonstrated that a grade-III PS is only seen in the absence of both the ACL and ALL in vitro. Other studies have also indicated the importance of the iliotibial band (ITB) 19 20 and the anterolateral structures in restraining the IR. 21 22 According to Geeslin et al., 19 restriction of the PS in the ACL-deficient knee is attributed to the ALL and the Kaplan fibers, and bending angles between 60° and 90°, with the section of Kaplan fibers leads to the highest IR.

The anterolateral structures are frequently injured during ACL ruptures. 23 These combined injuries may result in an increased anterolateral rotational laxity 17 23 ; therefore, in some cases of ACL reconstructions, residual instability may remain. 20 24 25

Lateral extra-articular tenodesis has a better lever arm than that provided by the classic ACL reconstruction, 20 having better control of the internal tibial rotation and the PS, 19 23 26 27 being probably required to restore better stability in more severe cases. Numerous authors argue that the addition of an extra-articular procedure to an ACL reconstruction significantly reduces the prevalence of residual PS, allowing patients to return to activities earlier with a better subjective outcome. 28

Several studies have shown that the anterolateral structures of the knee act as major restraints to the IR of the knee, 29 working in synergy with the ACL, 30 and that the PS phenomenon seems to be associated with injury in these structures. 19 26 Therefore, by taking these findings into consideration, we may deduce that when a significant increase in the static IR is noted in clinical practice, a possible undetected lesion to the anterolateral structures of the knee might occur. Yet, in this setting of increased knee IR, it would be necessary to add an extra-articular procedure to an ACL reconstruction to restore native knee kinematics.

This study has some limitations, such as non-randomization, a single evaluating surgeon, and manual testing, which requires greater care to maintain torque and similar bilateral foot contact points. However, this approach has the advantage of being similar to everyday life, mainly in underdeveloped countries where access to navigation technology is unfeasible in daily practice. Therefore, the current paper can be more directly applied to the everyday clinical practice.
