# Supplementary material for: Must a Knee with Anterior Cruciate Ligament Deficiency and High-grade Pivot Shift Test Present an Increase in Internal Rotation?
Source: Rev Bras Ortop (Sao Paulo). 2024 Mar 21;59(1):e82–7. doi: 10.1055/s-0044-1779316 (PMC10957268; doi:10.1055/s-0044-1779316)
Supplement: Related texts

# Research article: Um joelho com deficiência de ligamento cruzado anterior e alto grau no teste de *pivot shift* deve apresentar aumento de rotação interna? *

**Keywords:** ligamento cruzado anterior, instabilidade articular, fenômenos biomecânicos

## Abstract

**Objetivo:** A ruptura do ligamento cruzado anterior (LCA) é uma das lesões mais comuns em atletas e está frequentemente associada a danos nas estruturas anterolaterais. Esta combinação de lesões apresenta-se clinicamente como um teste de *pivot shift* de alto grau. A hipótese deste estudo é que pacientes com deficiência de LCA e teste de *pivot shift* de alto grau tenham aumento da rotação interna (RI) do joelho.

**Métodos:** Vinte e dois pacientes foram testados. Após raquianestesia efetiva, foram realizados dois testes com o paciente em posição supina. Primeiro, o teste de *pivot shift* bilateral foi realizado manualmente e seu grau foi registrado. Em seguida, com o joelho flexionado a 90 graus, o examinador traçou a projeção do pé em posição neutra e em RI máxima e mediu o ângulo de RI a partir dos eixos construídos entre o ponto central do calcanhar e o hálux.

**Resultados:** Joelhos com deficiência de LCA apresentam delta da RI média estatisticamente significativo de 10,5 graus entre os grupos sem ajuste de idade e de 10,6 graus com ajuste de idade.

**Conclusões:** Joelhos com deficiência do LCA e teste de *pivot shift* de grau I não apresentam aumento da RI em relação aos joelhos com LCA íntegro. Joelhos com deficiência do LCA e teste de *pivot shift* de graus II e III apresentam RI em comparação aos joelhos saudáveis.

## Introdução

A ruptura do ligamento cruzado anterior (LCA) é uma das lesões mais comuns em atletas. 1 Sua reconstrução é um dos procedimentos ortopédicos mais realizados e apresenta algumas complicações pós-operatórias, como controle rotacional insatisfatório, com taxa de sucesso entre 69 e 95%. 2 3 Esse restauro inadequado da biomecânica faz com que um número significativo de pacientes não retorne ao seu nível e/ou tipo de atividade física anterior; 4 além disso, esses indivíduos apresentam maior risco de nova falha ligamentar. 5

Como demonstrado por Terry et al., 6 a grande maioria dos joelhos com ruptura do LCA também tem lesão nas estruturas laterais associadas ao trato iliotibial (TIT). O ligamento anterolateral (LAL) atua na estabilidade rotacional do joelho com lesão do LCA; 7 assim, a lesão associada desses ligamentos aumenta a rotação interna (RI) do joelho e o grau de *pivot shift* (PS), 8 também sendo apontada como causa de falhas na reconstrução do LCA. 8

O teste PS positivo é um importante indicador de instabilidade rotacional do joelho. 9 Algumas meta-análises mostraram uma alta taxa de pacientes com resultado persistentemente positivo no teste PS no período pós-operatório. 10 11 12 Em uma revisão sistemática, Mohtadi sugere uma prevalência de 19% de teste PS de grau II ou superior após a reconstrução do LCA; 11 outros estudos mostram que este teste permanece positivo em mais de 30% dos casos e que esta instabilidade provoca lesões secundárias do menisco e da cartilagem. 13 14

Diversos estudos em cadáveres demonstraram que, na presença de lesão concomitante do LCA e das estruturas anterolaterais, há aumento do grau de PS e RI passiva do joelho em ângulos de flexão acima de 30°; 8 entretanto, faltam estudos clínicos que explorem esses dois testes juntos. O presente estudo tem como objetivo correlacionar o grau de PS e a RI do joelho em pacientes com lesão do LCA. O teste PS é subjetivo e não apresenta boa precisão ou reprodutibilidade. 15 A medida da RI do joelho a 90° de flexão é um teste objetivo do exame físico e, portanto, apresenta provável baixa variabilidade intra e interobservador. Assim, pretendemos estabelecer um exame clínico mais confiável, com reprodutibilidade e padronização mais fáceis para o diagnóstico de instabilidade rotacional anterolateral do joelho.

A hipótese deste estudo é que pacientes com deficiência do LCA e alto grau no teste PS apresentem aumento da RI do joelho.

## Métodos

## Estudo transversal

Todos os procedimentos realizados em estudos com participantes humanos estavam de acordo com os padrões éticos e foram aprovados pelo comitê de ética em pesquisa do Hospital de Clínicas de Porto Alegre (CAAE número 09548118.9.0000.5327). Um estudo piloto foi realizado para cálculo amostral, com nível de significância de 5%, poder de 90% e tamanho de efeito de 1,5 desvios-padrão (DPs) entre grupos, obtendo-se um mínimo de 10 pacientes por grupo. Houve diferença na RI média de 8,5 entre o joelho com lesão e o joelho sadio entre os grupos I (PS 1; RI média = 9,83, DP = 3,25, n = 6) e II (PS 2 e 3; RI média = 1,33, DP = 2,06, n = 6).

Dentre os pacientes com maturidade óssea completa e deficiência crônica do LCA que seriam submetidos à reconstrução ligamentar em Porto Alegre, RS, Brasil, entre agosto de 2019 e setembro de 2020, 22 foram analisados. Nenhum joelho apresentou lesões na raiz meniscal posterior ou lesão na rampa meniscal. Dos 22 pacientes incluídos no estudo, 15 apresentavam lesões meniscais (68,18%). Desses 15 pacientes, 6 foram designados para o grupo II: 3 com lesão no menisco lateral, 1 com lesão no menisco medial, 1 com lesão em ambos os meniscos e 1 com lesão no menisco lateral + lesão condral de grau IV menor que 1 cm ^2^ . No grupo I, nove pacientes apresentavam lesões meniscais: quatro tinham lesões no menisco lateral, quatro tinham lesões no menisco medial e um tinha lesões em ambos os meniscos.

Onze pacientes (1 mulher e 10 homens) com PS de grau I foram alocados no grupo 1, enquanto 6 pacientes com PS de grau II e 5 pacientes com PS de grau III foram incluídos no grupo 2 (2 mulheres e 9 homens) ( Fig. 1 ). A média de idade dos pacientes foi de 22 anos. A amplitude de movimento mínima esperada para todos os joelhos estava entre 0° e 130°. Os pacientes responderam um questionário sobre idade, sexo, tempo de lesão, presença de alguma doença sistêmica ou lesão em membros inferiores. Pacientes com histórico de lesão prévia, cirurgia ou doenças neurológicas do joelho ou membro inferior, bloqueio do joelho, doença reumatoide ou outra doença inflamatória das articulações, malformação congênita dos membros inferiores que possa influenciar a rotação da perna ou do pé ou artrose significativa foram excluídos.

**Fig. 1:** Proporção de homens e mulheres nos grupos I e II.

Após a raquianestesia, dois exames foram realizados com os pacientes em posição supina. Primeiro, o teste PS bilateral foi realizado manualmente e seu grau foi registrado de acordo com a classificação determinada pelo *International Knee Documentation Committee* (IKDC). 16 Em seguida, enquanto um auxiliar mantinha a coxa do paciente imobilizada, o joelho era flexionado a 90° e o pé era apoiado sobre uma mesa rígida, o examinador traçou a projeção do pé em posição neutra e em RI máxima, girando o calcanhar sobre seu próprio eixo, utilizando o polegar para fazer um fulcro na face medial do calcâneo e os demais dedos para pressionar a face lateral da base do quinto metatarso ( Fig. 2 ). O ângulo de RI foi medido a partir dos eixos construídos entre o ponto central do calcanhar e o hálux. A RI máxima foi obtida quando o aumento da carga não gerou mais movimento.

**Fig. 2:** Protocolo de rotação interna: a: pé em posição neutra; b: pé em rotação interna máxima; c: dedos pressionando a face lateral da base do quinto metatarso; d: polegar fazendo fulcro na face medial do calcâneo; e: eixos construídos entre o ponto central do calcanhar e o hálux (pé em posição neutra e em rotação interna máxima).

Todos os exames foram realizados bilateralmente pelo mesmo examinador enquanto o paciente estava anestesiado, seguindo o mesmo protocolo para todos os indivíduos e antes de qualquer incisão cirúrgica. Em cada paciente, a RI do joelho sem lesão foi considerada normal.

## Análise estatística

As variáveis foram descritas por médias e desvios-padrão. A comparação de médias foi baseada no teste t de Student. O controle do efeito da idade utilizou a análise de covariância (ANCOVA). O nível de significância adotado foi de 5% ( *p* < 0,05) e as análises foram realizadas no programa IBM SPSS *Statistics for Windows* , versão 21.0 (IBM Corp., Armonk, NY, EUA).

## Resultados

A idade média dos participantes foi de 22 anos, sendo 25,4 no grupo 1 e 20,4 no grupo 2. O participante mais jovem tinha 16 anos e o mais velho tinha 36 anos. O grupo 1 apresentou média de idade significativamente maior que o grupo 2 (25,4 ± 5,4 *vs* 2,4 ± 2,5; *p* = 0,012) ( Fig. 3 ). Dentre os 22 participantes, 3 eram do sexo feminino e 19 do sexo masculino ( Fig. 1 ). A RI média dos joelhos com LCA preservado foi de 30,72° (18° a 48°; n = 22). A RI média dos joelhos com lesão do LCA no grupo 1 foi de 30° (21° a 47°; n = 11). A RI média dos joelhos com lesão do LCA no grupo 2 foi de 43,36° (36° a 54°; n = 11).

**Fig. 3:** Idade média dos participantes nos grupos I (PS 1) e II (PS 2 e 3).

A Tabela 1 e a Fig. 4 mostram a comparação entre os grupos em relação ao delta de RI. Observamos uma diferença média de 10,5° entre os grupos na ausência de ajuste por idade e de 10,6° após o ajuste por idade. As diferenças foram estatisticamente significativas ( *p* < 0,001) nas duas condições.

**Tabela 1:** Comparação do delta de rotação interna do joelho nos dois grupos

	Média ± desvio-padrão		Diferença (IC 95%)	Valor de *p*	Diferença ajustada * (IC 95%)	Valor de *p*
	PS 1	PS 2/3				
**Delta RI (°)**	0,7 ± 1,0	11,2 ± 4,1	10,5 (7,8–13,1)	< 0,001	10,6 (7,3–13,8)	< 0,001

Abreviaturas: IC, intervalo de confiança; PS, *pivot shift* ; RI, rotação interna.

* Ajustada por idade pela análise de covariância (ANCOVA).

**Fig. 4:** Rotação interna média em participantes dos grupos I (PS 1) e II (PS 2 e 3).

## Discussão

Nossos resultados confirmam a hipótese primária: joelhos com deficiência do LCA e maior instabilidade anterolateral (PS de grau II ou III) apresentam aumento da RI em relação ao joelho contralateral com LCA íntegro. Por outro lado, em joelhos com deficiência do LCA e menor instabilidade anterolateral (PS de grau I), não houve aumento da RI em relação ao joelho contralateral com LCA íntegro.

O objetivo deste estudo foi comparar o aumento da RI entre os joelhos do grupo PS 1 e do grupo PS 2 ou 3. Verificou-se que pacientes com PS 2 ou 3 apresentam maior aumento na RI do que pacientes com PS 1. À cinemática, houve alterações diferentes na estabilidade rotacional entre os grupos estudados, corroborando nossa hipótese. Portanto, o aumento da RI do joelho tem importância clínica significativa.

Os primeiros pesquisadores a apontarem a importância do ligamento anterolateral (LAL) foram Claes et al., 17 que concluíram que o LAL tem grande importância como estabilizador da RI em joelhos com ângulo de flexão maior que 35°. Estes autores também observaram que o aumento da flexão diminui a participação do LCA nesta função. Rasmussen et al. demonstraram que a deficiência de LCA/LAL provocou aumentos significativos na RI estática, na translação do plano axial e na RI durante o PS simulado em comparação aos estados intacto e com deficiência de LCA em todos os ângulos de flexão. A deficiência do LCA causou aumentos significativos na RI em 0° a 45° de flexão do joelho em comparação ao estado intacto. 18 Bonanzinga et al. 5 mostraram que o LAL desempenha um papel significativo no controle da RI estática e do PS no joelho com deficiência de LCA. Monaco et al. 14 demonstraram *in vitro* que o PS de grau III só é observado na ausência do LCA e do LAL. Outros estudos também indicaram a importância da banda iliotibial (BIT) 19 20 e das estruturas anterolaterais na restrição da RI. 21 22 Segundo Geeslin et al., 19 a restrição do PS no joelho com deficiência do LCA é atribuída ao LAL e às fibras de Kaplan e que em ângulos de flexão entre 60° e 90°, a secção das fibras de Kaplan aumenta a RI.

A lesão das estruturas anterolaterais é frequentemente associada às rupturas do LCA. 23 Essas lesões combinadas podem aumentar a lassidão rotacional anterolateral 17 23 ; portanto, pode haver instabilidade residual após algumas reconstruções do LCA. 20 24 25

A tenodese extra-articular lateral possui braço de alavanca melhor que o proporcionado pela reconstrução clássica do LCA, 20 tendo melhor controle da RI da tíbia e do PS, 19 23 26 27 sendo provavelmente necessária para melhor restauro da estabilidade em casos mais graves. Numerosos autores argumentaram que a adição de um procedimento extra-articular a uma reconstrução do LCA reduz significativamente a prevalência de PS residual, permitindo que os pacientes retornem às atividades mais cedo com melhor desfecho subjetivo. 28

Diversos estudos demonstraram que as estruturas anterolaterais do joelho atuam como grandes restrições à RI articular, 29 trabalhando em sinergia com o LCA, 30 e que o fenômeno de PS parece estar associado a lesões nessas estruturas. 19 26 Portanto, considerando esses achados, podemos deduzir que a observação de um aumento significativo da RI estática na prática clínica pode estar associada a uma possível lesão não detectada nas estruturas anterolaterais do joelho. No entanto, neste cenário de aumento da RI do joelho, seria necessário adicionar um procedimento extra-articular a uma reconstrução do LCA para restauro da cinemática nativa do joelho.

Este estudo tem algumas limitações, como a ausência de randomização, a existência de um único cirurgião avaliador e a realização de testes manuais, o que requer maior cuidado para manter o torque e os pontos de contato bilaterais semelhantes nos pés. Contudo, esta abordagem tem a vantagem de ser semelhante à vida cotidiana, principalmente em países subdesenvolvidos em que o acesso à tecnologia de navegação é inviável na prática diária. Portanto, o presente artigo pode ser aplicado de forma mais direta à prática clínica cotidiana.
